# Are We Any Closer to Understanding How Chronic Pain Develops? A Systematic Search and Critical Narrative Review of Existing Chronic Pain Vulnerability Models

**DOI:** 10.2147/JPR.S411628

**Published:** 2023-09-14

**Authors:** Ariane Delgado-Sanchez, Christopher Brown, Manoj Sivan, Deborah Talmi, Christiana Charalambous, Anthony K P Jones

**Affiliations:** 1 Division of Human Communication, Development, and Hearing, University of Manchester, Manchester, UK; 2 Institute of Population Health, University of Liverpool, Liverpool, UK; 3 Leeds Institute of Rheumatic and Musculoskeletal Medicine, University of Leeds, Leeds, UK; 4 Department of Psychology, University of Cambridge, Cambridge, UK; 5 Department of Mathematics, University of Manchester, Manchester, UK

**Keywords:** vulnerability, evaluation, theory, conceptual model

## Abstract

Identifying biopsychosocial factors underlying chronic pain vulnerability is essential for the design of preventative efforts. Multiple chronic pain vulnerability models exist, however, there is a lack of comprehensive evaluation of these models in the literature, potentially due to the lack of guidelines that specify the criteria by which these types of work should be assessed. In this work, we created evaluation criteria (based on the general goals of conceptual models), and we then used them to critically review the chronic pain vulnerability models available in the current peer-reviewed literature (identified through a systematic search). Particularly, we evaluated the models on the basis of conceptual clarity/specificity of measures, depth of description of aetiological and mechanistic factors, use of a whole system approach, and quality of the evidence associated with the models. We found nine conceptual models that have been explored in detail (eg, fear avoidance model, diathesis-stress model). These models excel at clarity and are supported mostly by self-report evidence of a psychological nature (anxiety sensitivity, pain catastrophizing, etc.), but provide little explanation of mechanistic and aetiological factors. In the future, models could be improved by complementing them with proposals from other models and exploring potential causal factors and mechanisms maintaining the condition. This task could be carried out through prospective cohort studies, and computational approaches, amongst others.

## Plain Language Summary

Several theories explain why some people are more vulnerable to develop chronic pain; however, there is no previous work that compares these theories or evaluates their quality. After evaluating these theories, we concluded that they are very good at identifying what psychological characteristics make someone vulnerable to develop chronic pain. However, they lack an explanation regarding what makes people develop those characteristics or how they lead to pain. We propose ways in which the different theories could be complemented.

## Introduction

Chronic pain is defined as “persistent or recurrent pain lasting longer than 3 months”.^1^ The ICD-11 divides pain into two main categories: primary pain (chronic pain in which there is no identifiable underlying cause or in which symptoms are out of proportion to the identified cause) and secondary pain (pain is a consequence/symptom of another condition).^2^ Chronic pain is an important public health issue affecting between 35% and 51.3% of the UK population^3^ and 19% of the European population.^4^ Although still not fully understood, the factors influencing the development of vulnerability – the relative risk to develop chronic pain – are not completely unknown. Several conceptual models of chronic pain vulnerability have gathered supporting evidence.

We define conceptual models as “visual or written products that explain, either graphically or in narrative form, the main things to be studied—the key factors, concepts, or variables—and the presumed relationships between them”.^5^ Models or theories are key elements in the development of research and intervention. In fact, theoretical illiteracy has been pointed out as an important obstacle for the progress of scientific advancement.^6^ Nevertheless, although it is clearly important to have good theories, there are no specific guidelines to assess the quality of conceptual models. In this work, we used the literature regarding the use of conceptual models and the proposals made regarding what makes a good theory to develop evaluation criteria for conceptual models. We then used these criteria to assess the most prominent chronic pain vulnerability models.

## Methods

The current review analysed and evaluated the current conceptual models of chronic pain vulnerability. To begin with, we explored the literature regarding the use of conceptual models to identify the criteria that should be used to evaluate this type of scientific work. We then used the outputs of this research to develop specific evaluation criteria for conceptual models. Note that the developed criteria are not exclusive to chronic pain and could be applicable to any conceptual model exploring a health condition or psychological concept. Nevertheless, in this specific work, we used them to evaluate the quality of conceptual models exploring chronic pain vulnerability.

Current chronic pain vulnerability models were identified by performing a systematic search on Web of Science, PubMed and PsycINFO for the terms “chronic pain” and “vulnerability” and (“model” or “theory” or “framework”). These searches lead to 270, 349 and 239 results, respectively, in each of the databases. The decision to include the three terms (model, theory and framework) was taken based on the interchangeable use that is given to these three concepts in the literature. Furthermore, models were also included based on knowledge of experts in the field.

In the literature search, after deleting duplicates, the titles and abstracts of the papers were analysed. Models were included in this work if they proposed (1) an explanation for the aetiology of primary pain (ie, pain is the main disorder)^2^ or (2) an explanation for inter-individual differences in chronic pain severity or pain intensity in secondary pain (ie, pain is a consequence of another disorder).^2^ Empirical studies and reviews that presented the following characteristics were excluded: articles in which chronic pain was used as the predictor and not the outcome; articles in which pain was not treated as the main symptom but just one of the symptoms in another condition that is being studied; articles in which the quality of life/pain intensity was assessed in population who had already developed chronic pain; and articles in which the model refers to a statistical model only as a methodology, animal models, treatment models or drug use control/trajectory models. Finally, papers that were not available in English or Spanish were also excluded. The remaining articles were inspected to identify the disease vulnerability models present in the literature.

**Figure 1:**
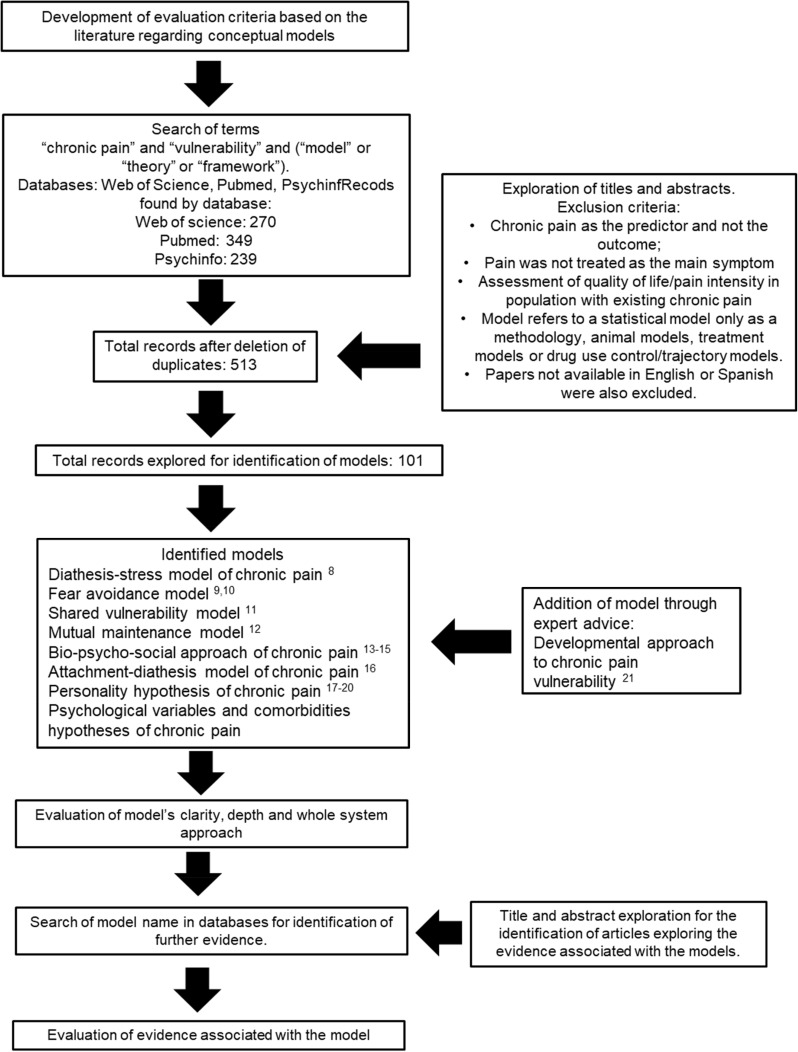
Flowchart of review process.^8–21^

Once the models were identified, a further search of the literature was performed with each one of them (by searching the model name in the three databases mentioned above) to gather further information on their proposal and associated evidence. We explored the titles and abstracts to identify the papers that explored the evidence associated with the models of interest and these were used to perform the subsequent evaluation. Since one of the goals of this work was to evaluate quality of the evidence associated with the models, we included papers from all different levels of evidence and then assessed them in the evidence section of the evaluation criteria (see Table 1). A flowchart of the methods can be found in Figure 1.

**Table 1:** Model Evaluation Criteria

	Model Evaluation Criteria
**Clarity or specificity**	Whether the model presented specific and measurable variables was evaluated. This criterion was included in order to assess the clarity and applicability of the model to identify patients or potential patients at greater risk (of developing primary pain or intense secondary pain). It was assumed that if specific variables could not be obtained from the model, its use in clinical settings would be limited. Clarity was measured by answering one question
	*1. Does the model propose a specific set of variables that can be measured?*
**Depth of aetiological and mechanistic factors**	This criterion was used to measure whether the model hypotheses explored in depth the origins of the proposed set of variables/characteristics. Note this does not mean whether the models explain in detail the proposed variables. Specifically, the criterion of depth was used to assess whether the model identified aetiological or risk factors (environmental, genetic, epigenetic) and mechanistic (biological, psychological and social) factors that could be used to develop or research future intervention and prevention procedures. In other words, the criteria of depth evaluated if the model provided further explanation as to how the conglomerate of variables identified was created and maintained. Depth was measured by answering two questions
	*2. Does the model propose the aetiological factors that lead to the development of the proposed set of variables?*
	*3. Does the model provide a mechanistic explanation of chronic pain vulnerability?*
**Whole system approach**	This criterion was used to assess whether the model provided a representation of the multifactorial nature of the pain phenomenon by taking a whole system approach (integrating biological, social, ecological and psychological components). The whole system approach was measured by answering 1 question
	*4. Does the model take a whole system approach?*
**Evidence**	This criterion was used to assess the evidence-based support of the model. In this section of the model evaluation tables, first the evidence regarding the model will be presented. Furthermore, a label will be provided to assess the quality of the evidence based on the study designs described in the Oxford Centre for Evidence Based Medicine criteria for evidence levels and grades.^7^ The evidence levels associated with each study based on these criteria are the following: ● Level I: Systematic review of Randomized Controlled Trials (RCTs) or individual RCTs ● Level II: Cohort studies. ● Level III: Case-control studies ● Level IV: Case series studies ● Level V: Expert opinion Based on these levels of evidence the grade of recommendation associated with the models can be evaluated based on the following guidelines:^7^ ● *A: consistent level 1 studies* ● *B: Consistent level 2 or 3 studies* ● *C: Consistent level 4 studies* ● *D: level 5 evidence or inconsistent results from studies at any level.* Evidence was measured by answering 1 question
	*5. What is the level of evidence provided to support this model?*

## Results

### Evaluation Criteria for Conceptual Models

Evaluation criteria to assess the quality of the models were developed based on the principles and objectives of conceptual models in general. Overall, different authors have proposed that conceptual models should be tools to guide practice and research.^22^,^23^ It has also been proposed that theories should provide an explanation for the causal and maintenance factors of a disease^6^ and that their best use is achieved when they integrate socio-ecological and biological components.^23^ Based on these proposals regarding the objectives of models and theories, we concluded that a model’s quality should be evaluated based on the following three principles: (1) its usefulness in practice, (2) its ability to direct research to the insight into causal and maintenance factors and (3) its use of a whole system approach (integration of biological, ecological, psychological and social components). Furthermore, based on the risk of dogmatic acceptance of existing theories pointed out in the literature^6^ we proposed a fourth principle, 4 - the empirical support associated with the model.

In this work, we developed evaluation criteria to assess conceptual models based on the key objectives associated with these types of theories. In order to evaluate the usefulness of a theory in practice (principle 1) we assessed whether specific measurable factors were proposed that could be of use for clinicians. To measure the ability to direct research into causal and maintenance factors (principle 2) we evaluated whether the model proposed any causal or risk factors linked to the development of the main variables and whether it presented a mechanistic explanation. It is worth clarifying that we understand mechanism both as a “complex arrangement of entities and activities, organised in such a way as to be regularly or predictably responsible for the phenomenon to be explained” and a “spatio-temporal pathway along which certain features are propagated from the starting point to the end point”.^24^ The whole system approach was evaluated by analysing whether the model integrated social, ecological, psychological and biological components. Finally, the evidence supporting the model was evaluated based on the Oxford Centre for Evidence-based Medicine Levels of Evidence (OCEBM).^7^ All of these points were formalised in the evaluation criteria presented in Table 1 and subsequently used to assess the identified models.

### Current Models for Chronic Pain Vulnerability

The following models were identified through the review: the diathesis-stress model of chronic pain,^8^ the fear avoidance model,^9^,^10^ the shared vulnerability model,^11^ the mutual maintenance model,^12^ the bio-psycho-social approach of chronic pain^13–15^ the attachment-diathesis model of chronic pain,^16^ the personality hypothesis of chronic pain,^17–20^ and the psychological variables and comorbidities hypotheses of chronic pain. The following model was added as a consequence of experts’ review: the developmental approach to chronic pain vulnerability.^21^

#### Diathesis-Stress Model of Chronic Pain^11^

This model was created with the objective of explaining the development of disability and chronic pain after a traumatic experience, when there is no tissue damage that would explain the level of disability. Turk^8^ proposes that when faced with a traumatic event (stress), the likelihood of a patient developing chronic pain could be influenced by predisposing cognitive and behavioural factors (diathesis). Particularly, this model gives special attention to Anxiety Sensitivity (AS) as a predisposing factor. “As is the fear of arousal-related sensations, arising from beliefs that certain sensations have adverse consequences such as death, insanity, or social rejection”.^25^ Turk^8^ proposes that high levels of this construct will lead to an increased fear of pain, which in turn results in a higher anticipation of pain and higher pain perception. Furthermore, the fear of pain will make patients avoid behaviours that could cause an unpleasant sensory experience and, therefore, predispose them to more fear and disability.

Moreover, people with high AS will present hypervigilance towards their bodily sensations. In this way, when faced with a threatening event (such as a car accident), their attentional bias may make them interpret innocuous bodily sensations as abnormal or even painful, and establish a causal attribution with the threatening event. This interpretation of bodily sensations will in turn lead to higher fear and anticipation of pain.

High AS is linked with increased catastrophizing within the model. Catastrophizing has several definitions in the field of pain research.^26^,^27^ Turk^8^ clarifies that in their model catastrophizing is defined as a coping mechanism characterised by negative self-statements, and overly negative thoughts and ideas about the future.^8^ The model states that increased catastrophizing once again results in increased fear of pain.

Finally, the concepts of self-efficacy and operant conditioning are also included in the proposal. As previously stated, the final result of the psychological and cognitive diathesis is an increased avoidance of behaviours that are feared to cause pain. In this way, whilst operant conditioning feeds back the maladaptive cognitions and beliefs; higher self-efficacy leads to a lower avoidance of behaviours perceived as threatening.^8^

Little research has focused on empirically testing the model; nevertheless, the existing few attempts have had positive results.^28^ In general, most studies have found a correlation between most of the proposed variables with pain^29^,^30^. Nevertheless, although the different elements of the model seem to be related to pain, there is still an open debate regarding the hierarchy of importance of the different psychological factors. For instance, anxiety sensitivity, which is given a central role in this and other models, has failed to show an effect on pain when controlling for other variables.^29^ Future research should attempt to test the mediating factors and determine which variables are central for the development of chronic pain and which represent just a moderating or confounding effect.

Furthermore, although this model very clearly states the variables that should be measured to test vulnerability, it does not provide an explanation as to what leads to the development of this vulnerability. The model takes almost exclusively a psychological perspective, leaving out biological and social components. In order to improve the model and promote research in the area it would be prudent to expand the proposal to include both aetiological factors and bio-social correlates that could help present a clearer picture of the phenomenon.

A summary of the evaluation criteria results can be observed in Table 2, and the full application of the evaluation criteria on the model can be seen in Table 3.

**Table 2:** Summary and Comparison Table of the Current Chronic Pain Vulnerability Models

	Clarity	Depth		Bio-Psycho-Social Approach	Evidence
		Aetiology	Mechanisms		
Diathesis-stress model	Yes	No	Yes. Psychological.	No	Grade C
Fear Avoidance model	Yes	No	Yes. Psychological.	No	Grade B
Shared vulnerability and mutual maintenance	Yes	No	Yes. Psychological	No	Grade B
The biopsychosocial model/approach	No	Yes	Yes	Yes	Grade A
The Attachment-Diathesis Model	Yes	Yes	Yes. Psychological and social	No	Grade C
The Personality hypothesis	No	Yes	No	No	Grade D
Psychological variables and comorbidities	No	No	No	No	Grade B
Developmental approach to chronic pain vulnerability	No	Yes	Yes	Yes	Grade B

**Table 3:** Evaluation of the Diathesis-Stress Model of Chronic Pain^12^

Model Evaluation: Diathesis-Stress Model of Chronic Pain^12^	
**Clarity**	
*Does the model propose a specific set of variables that can be measured?*	
Yes	The variables of the model are clearly specified: trauma, anxiety sensitivity, fear of pain, catastrophizing, self-efficacy, escape/avoidance and disability.
**Depth**	
*Does the model propose the aetiological factors that lead to the development of the proposed set of variables?*	
No	Although the model provides a specific set of characteristics (eg anxiety sensitivity) that lead to a higher vulnerability to developing chronic pain when faced with trauma, it does not provide an explanation as to what is the cause of the variability of those factors.
*Does the model provide a mechanistic explanation of chronic pain vulnerability?*	
Yes	The model provides psychological, cognitive and behavioural mechanisms that lead to the development of chronic pain.
**Whole system approach**	
*Does the model take a whole system approach?*	
No	The model presents a psychological/cognitive focus only.
**Evidence**	
*What is the level of evidence provided to support this model?*	
Grade C	Few investigations have been performed to study the veracity of the present model. However, the ones that have done so have obtained supporting results. For instance, one cross-sectional study corroborated the full model through Structural Equation modelling.^28^ Evidence regarding the significant effect on pain of the different variables proposed in the model such as pain catastrophizing (with a cross-sectional design)^29^ has been gathered. Evidence of the effects of the variables proposed in the model has been observed. For instance, the effect of AS on pain through an increase of fear of pain has been observed in another cross-sectional study.^31^ Furthermore, a Structural Equation Modelling approach showed an effect of anxiety sensitivity and pain catastrophizing on fear of pain (through an increase in hypervigilance).^32^ In this model, a central role is given to the variable of AS; nevertheless, some pain studies with cross-sectional designs have failed to find an influence of this variable when controlling for other factors.^29^ Furthermore, a cohort study showed that although AS had an effect on the pain trajectory after traumatic injury, the nature of the effect was not completely clear since AS showed to increase the chances of acute pain but high AS at the time of traumatic injury was also a predictor of for recovery from acute pain (lack of chronification).^30^ Finally, another cohort study failed to find the effect of AS on pain. These results could be just an indication of the existence of other mediating variables and the need to use a proper statistical approach; nevertheless, with the current knowledge it is difficult to attribute causality and the proposed hierarchical effect. In summary, the effect of the variables proposed in the model on pain and some of the proposed relationships between the variables are supported by the evidence of mostly cross-sectional designs. However, some cross-sectional and cohort studies find conflicting evidence on the effect of AS Few attempts exist to test the full model and the level of evidence associated with it is mostly of level 4.

#### Fear Avoidance Model^9^,^13^

This model was created to explain the “exaggerated pain perception” observed in some chronic low-back pain patients.^9^ It is one of the most researched models in chronic pain vulnerability and it delves into very similar concepts as the ones presented in the Diathesis-Stress model; however, the current model has a central focus on the concept of fear of pain. In the original proposal, Lethem et al^9^ postulated that when faced with a painful experience, individuals could take two approaches based on their fear levels: confrontation (more likely to occur with low fear) or avoidance (more likely to occur with high fear). These approaches would lead to opposite consequences. Confrontation would lead patients to mobilisation, calibration of the pain experience with the nociceptive input and effective rehabilitation. Meanwhile, avoidance would lead to reinforcement of the fear of pain and avoidant behaviour, physical consequences due to lack of movement and exaggerated pain perception. Furthermore, Lethem et al^9^ stated that fear of pain is mediated by the psychosocial context of each patient.

The general principle of the model stood the test of time (fear of pain leads to disability/chronicity) and subsequent proposals refined the originally proposed model. This way, some years later the model was applied to musculoskeletal pain.^10^ In this new postulation, authors included other elements that could be feeding into the fear of pain, such as pain catastrophizing and negative affect. Furthermore, they also included, as a consequence of fear of pain, the increase in hypervigilance to one’s bodily sensations.

These two versions of the fear avoidance model are not the only ones that have been postulated. Several researchers have proposed small alterations to this model, such as the inclusion of resilience as a protective factor influencing fear.^33^

This model is probably the most widespread model of chronic pain vulnerability and has proven useful for many researchers and clinicians. Particularly, its predictiveness of disability has been corroborated by the literature on multiple occasions.^31^,^33–36^ Nevertheless, although the general principle of the model seems to be solid, conceptual details and the specific effect of different variables have shown less clear results. For instance, some research has encountered a lack of effect of certain variables such as anxiety sensitivity;^37^ other investigations have found that the relationships between fear and pain are moderated by factors not included in the model,^38^,^39^ and finally, some researchers have encountered that adding new factors such as resilience to the model could improve its predictive accuracy.^33^ Taking all of this into account, it is important for future research to explore the exact mechanisms that could be leading the correlation between fear and pain in order to provide some clarity to the model’s proposals and better guide the design of interventions based on the principles defined by the model.

Furthermore, a higher focus on the aetiological factors that lead fear of pain would be beneficial. Some proposals have postulated the idea of prior experience with pain being a potential cause of the vulnerability;^9^ future research could explore this in more depth, as well as analysing other potential influencing factors. Finally, as with the diathesis-stress model, not much detail is provided regarding the bio-social correlates of the psychological variables proposed, which would be a good addition to the theory.

A summary of the evaluation criteria results can be observed in Table 2, and the full application of the evaluation criteria on the model can be seen in Table 4.

**Table 4:** Evaluation of the Fear Avoidance Model^13^,^14^

Model Evaluation: Fear Avoidance Model^13^,^14^	
**Clarity**	
*Does the model propose a specific set of variables that can be measured?*	
Yes	Although different versions of the model may present certain differential elements, the central factors of the model (fear of pain, avoidance or confrontation) are specified with clarity.
**Depth**	
*Does the model propose the aetiological factors that lead to the development of the proposed set of variables?*	
No	Some versions of the model mention aetiological factors such as previous experience with pain.^9^ Nevertheless, this is not the main focus of the model, this model is mostly centred on explaining the behavioural consequences of pain (disability), more so that the origins of pain.
*Does the model provide a mechanistic explanation of chronic pain vulnerability?*	
Yes	The model provides psychological, cognitive and behavioural mechanisms that lead to the development of chronic pain.
**Whole system approach**	
*Does the model take a whole system approach?*	
No	The model provides certain references to biological components like physical effects of avoidance, nevertheless the main focus of the model is on the psychological/cognitive components.
**Evidence**	
*What is the level of evidence provided to support this model?*	
Grade B	Numerous research attempts have been performed to study the validity of this model mostly with favourable results. A meta-analysis showed a medium to large effect size in the association between fear of pain and other factors of the model and a medium effect size in the association between fear of pain and pain intensity.^40^ Another meta-analysis showed a medium to small effect size on the effect of fear of pain on pain intensity^39^ Cross-sectional and cohort evidence supports the predictive value of fear avoidance on disability.^31^,^33–36^ Other relationships established in the model have also been tested through cross-sectional studies, for instance the correlation between hypervigilance and fear of pain.^32^ Aside from the evidence supporting the proposals of the original model, cohort research has shown that the model can improve its predictive accuracy through the inclusion of new variables such as pain catastrophizing or resilience.^33^ Some studies have found contradicting evidence regarding the psychosocial components that could be affecting disease progression. For instance, no effect of AS was observed in a disability development cohort study.^37^ Furthermore, that same study failed to find an effect of fear avoidance on the disability trajectory and only found an effect on pain. Some cross-sectional studies and meta-analyses have found that other variables like age, pain localization, etc. moderate the relationship between fear and pain.^38^,^39^ Although studies have shown that the model can be improved by including new elements and some contradicting evidence exists regarding the influence of some factors, the central proposals of the model are supported by cross-sectional, cohort and meta-analysis (of cross-sectional studies) evidence. Most of the evidence can be classified between levels 2 and 4.

### Mutual Maintenance^12^ and Shared Vulnerability Models^14^

These models were built to explain the comorbidity found between Chronic Pain and Post Traumatic Stress Disorder (PTSD). The shared vulnerability model proposed that the high co-occurrence of this disorders could be due to the same factors leading the vulnerability to chronic pain and PTSD. Particularly, the authors consider that AS may have a central role in increasing the vulnerability to both pathologies; therefore, someone with high AS would be more likely to develop both chronic pain and PTSD explaining part of the comorbidity between these two.^11^ Furthermore, in the Mutual Maintenance Theory, the authors propose that the comorbidity between these two disorders is a consequence of a feedback between several common or interacting symptoms:^12^

1. Attentional bias: both chronic pain and PTSD are proposed to be characterized by an increased attention to stimuli related to pain or the traumatic event respectively. Pain could be a reminder of the trauma in PTSD and therefore an increased attention to those stimuli may result in increased pain.
2. Anxiety Sensitivity is proposed as a common symptom in both disorders that increases catastrophizing and consequently the likelihood of interpreting the arousal characteristic of PTSD as potentially threatening stimuli.
3. Reminders of Trauma: As mentioned before, pain could be a reminder of the trauma which would increase arousal. Due to the proposed attentional bias in patients, the consequent increase in arousal and pain could result in an increase in fear and more avoidance.
4. Avoidance: The avoidance of behaviours that are feared to cause pain or may be a reminder of symptoms is proposed to prevent the extinction of negative reactions, and in turn favour the maintenance of both disorders.
5. Both disorders commonly co-occur with depression that will result in a decrease in activity that may serve to maintain both PTSD and chronic pain by giving way to avoidance of reminders of trauma and increasing disability respectively.
6. High anxiety levels are thought to reduce pain thresholds and tolerance.
7. Since dealing with the symptoms, such as catastrophic cognitions and intrusions, requires cognitive load, patients are thought to struggle to adopt cognitively active coping strategies to change these patterns.

In general, evidence exists to support the proposals of the models. The mutual maintenance model has received the most support,^41–43^ perhaps due to its higher level of detail that allows for its hypotheses to be studied in more depth. Nevertheless, it is important to mention that new research has found that the model could be improved by the inclusion of other variables such as sleep^44^ and mindfulness,^45^ indicating that the full phenomenon of the correlation between pain and PTSD is still a work in progress. It is important to consider that in the last decades since this model was proposed, research in the area of both PTSD and pain has grown exponentially, and therefore, an update of the model with the new knowledge from both fields would be beneficial. In order to improve the quality of the model, adding knowledge regarding bio-social factors would be particularly recommendable since, like in previously presented proposals, this model is mostly focused on psychological components.

When it comes to the shared vulnerability model, the results are not as solid. Once again, the model sets its focus on anxiety sensitivity, a variable that has shown some contradictory results in its association with pain.^29^,^37^ In order to improve the model, exploring other factors that lead to the vulnerability would be beneficial. Some investigations have already identified variables such as pre-injury health and social support,^46^ although more research is needed to corroborate these findings and identify further vulnerability factors. Furthermore, just like with the diathesis-stress and fear avoidance models, looking at the aetiological factors and mechanisms would be a beneficial addition to improve model quality.

A summary of the evaluation criteria results can be observed in Table 2, and the full application of the evaluation criteria on the model can be seen in Table 5.

**Table 5:** Evaluation of the Mutual Maintenance^12^ and Shared Vulnerability^11^ Models

**Model Evaluation: Mutual Maintenance^12^ and Shared Vulnerability^11^ Models**	
**Clarity**	
*Does the model propose a specific set of variables that can be measured?*	
Yes (with limitations)	In the case of the mutual maintenance model, a patient presenting one of the disorders and associated attentional symptoms constitutes vulnerability to the maintenance or chronification of the other disorder. In the case of the shared vulnerability model it is stated that variables predisposing to the two disorders could be the same, and it particularly addresses anxiety sensitivity as a possible predisposing factor; however, no further clear profile or list of suspected variables is provided.
**Depth**	
*Does the model propose the aetiological factors that lead to the development of the proposed set of variables?*	
No	Although some passing mention is made of possible effects of genetics the level of detail provided is low.
*Does the model provide a mechanistic explanation of chronic pain vulnerability?*	
Yes	The mutual maintenance model proposal is centred on explaining the mechanisms responsible for the mutual feedback between chronic pain and PTSD.
**Whole system approach**	
*Does the model take a whole system approach?*	
No	Although some biological mechanisms are included in the model (arousal), the model takes mostly a psychological or cognitive approach.
**Evidence**	
*What is the level of evidence provided to support this model?*	
Grade B	Although the shared vulnerability model has attracted less research than the mutual maintenance model, the comorbidity between chronic pain and anxiety disorders (not only PTSD) and the common predisposing variables (such as Anxiety Sensitivity) has provided some support to the proposal.^47–49^ Some aspects of the model, such as the effect of arousal leading to fear and re-experiencing and an effect on pain intensity, have been substantiated through cross-sectional studies that implemented structural equation modelling (although in the framework of the fear avoidance model).^50^ Several cohort studies have tested the mutual maintenance model with supporting results,^41–43^ although not all studies have found significant effects of all the variables of the model, for instance one study failed to find an effect of avoidance.^43^ New cross-sectional evidence has led to proposals regarding variables that could be influencing the relationship between disorders, such as sleep^44^ and mindfulness.^45^ Recent findings obtained through cohort studies suggest that the relationship between pain interference and PTSD could be asymmetrical. This way, people with PTSD would be more likely to develop pain interference but those with pain interference would not necessarily be at a higher risk to develop PTSD^46^ Studies have shown that the model can be improved by including new elements, furthermore, the evidence associated with some aspects of the model is stronger than others. However, in general, the central proposals of the model are supported by cross-sectional and cohort evidence. Most of the evidence can be classified between levels 2 and 4.

#### The Biopsychosocial Model/Approach of Chronic Pain

This model, instead of a formal chronic vulnerability model (like the ones presented above), could be classified more accurately as the application of the biopsychosocial approach^13^ to understanding chronic pain vulnerability.^51^ Perhaps, this is the reason why the focus on validating component factors of this model is quite variable. Some proposals are more dominated by the biological component with elements such as genetic, immune and endocrine factors being included.^14^ Other proposals give more attention to psychosocial factors and in the biological component they include elements such as comorbidities, sex and age.^15^ Nevertheless, in general terms, the biopsychosocial approach or model for chronic pain can be defined as those models or approaches that envision pain as a multidimensional, dynamic interaction among physiological, psychological, and social factors that reciprocally influence one another, resulting in chronic and complex pain syndromes.^52^

Although not always presented under the concept of the biopsychosocial model of pain, ample research exists on the different biological correlates of chronic pain vulnerability and their potential links with psychological and social components. For instance, one area of research that has gained a lot of attention links stressful life events with chronic pain vulnerability. It has been proposed that stressful life events increase individuals’ allostatic load (accumulation of stress consequences in the body) which leads to psychological, neuroendocrine, immune and brain alterations.^53^ Evidence exists linking early life stress with neuroendocrine alterations^54^ and high levels of inflammatory biomarkers,^55^ as well as evidence linking high inflammatory markers and sensitivity to pain.^56–58^ The exact mechanisms through which these alterations take place are still in the hypothetical stage^59^ but research efforts are being conducted to understand them. Moreover, other factors aside from stress have also been linked to these biological alterations such as diet^60^ or exposure to pollutants.^61^ Regarding the social components involved in the mechanisms described, research has linked some social factors, such as Socio Economic Status, with the experience of stressful events,^62^ higher consumption of pro-inflammatory diets^63^ and exposure to pollutants.^64^ Furthermore, other social elements have also gained attention in their relation with pain vulnerability and the associated psychological components. For instance, social support is an important predictor of disability.^65^

In summary, a lot of research is being developed in the area of the bio-psycho-social model of pain with supporting results. This model presents strengths unlike other models presented in this paper, mostly in providing aetiological and mechanistic explanations for chronic pain vulnerability and taking a whole-system approach. Nevertheless, this theory scores lower in one area in which other models excel: providing clear variables to measure by clinicians. Chronic pain is a multifactorial phenomenon that with our current knowledge is hard to fully summarize in a few variables – perhaps the reason why the other models opted to focus on just a few factors/a small part of the full phenomenon. Nevertheless, for clinical applications, it is important to understand what the key variables are in the complex cycle of chronic pain. Therefore, it would be beneficial for this model to evolve to identify the most clinically relevant variables that would make its application more feasible. Perhaps, research in which multiple different variables are measured and a reduction in dimensionality is performed through factor analysis would be recommendable. Another option would be to identify the mechanism(s) that link the different factors and use those as diagnostic and treatment targets (currently, research on metabolic and immune factors seems to be promising for this purpose^59^). Moreover, it is also important to note that the evidence supporting the model, such as that presented above, is often based on studies focusing on different components of the model separately. This way, there is a great amount of evidence linking psychological and biological components, social and psychological components or social and biological components. Nevertheless, studies that link the three components at once are less common. Consequently, the evidence oftentimes linked to the bio-psycho-social model could only be considered as partial support for the model instead of a strong corroboration of the model’s proposals.

A summary of the evaluation criteria results can be observed in Table 2, and the full application of the evaluation criteria on the model can be seen in Table 6.

**Table 6:** Evaluation of the Biopsychosocial Model/Approach of Chronic Pain

Model Evaluation: The Biopsychosocial Model/Approach of Chronic Pain		
**Clarity**		
*Does the model propose a specific set of variables that can be measured?*		
No		Differences in the variables that are part of the model can be seen from one publication to another.
**Depth**		
*Does the model propose the aetiological factors that lead to the development of the proposed set of variables?*		
Yes		Normally some aetiological factors are provided, nevertheless the detail given to these and the factors selected varies between proposals.^14^,^15^
*Does the model provide a mechanistic explanation of chronic pain vulnerability?*		
Yes		Varies between proposals. The detail given to these mechanisms and the factors selected varies between proposals.^14^,^15^
**Whole system approach**		
*Does the model take a whole system approach?*		
Yes		This is the application of the bio-psycho-social model to chronic pain vulnerability and therefore takes into consideration biological, psychological and social aspects of pain, which leads to a whole system approach.
**Evidence**		
*What is the level of evidence provided to support this model?*		
Grade A	Taking a bio-psycho-social approach to health has been supported by evidence since the first proposal of the model by Engel.^17^ Evidence supports the biological, psychological and social influences in pain.^14^,^66–71^ The use of biopsychosocial approaches in Randomized Clinical Trials has supported the validity of the model.^72^ In summary, evidence of different levels can be found supporting the general proposals of the model, including evidence of level 1.	

#### The Attachment-Diathesis Model of Chronic Pain^19^

Attachment theory is one of the most established and supported theories in psychology.^73^ This developmental theory states that an infant will develop an internal working model of relationships based on the early interactions they have with their primary caretaker. The internal working model is a mental representation or schema of oneself, others, and the world that will serve as a base for future appraisals, perceptions and behaviours. In general, individuals can be classified in three attachment styles: a secure attachment style, developed in those who encounter positive and sensitive interactions with their main caretaker; and two insecure attachment styles (insecure-avoidant and insecure-anxious ambivalent), for those who receive a less sensitive treatment from their caretakers.^74^

Since attachment style serves as the foundation of the appraisals and perceptions of an individual, the Attachment-Diathesis Model of Chronic Pain makes the following proposal: (1) When an individual is exposed to a painful stimulus the attachment style or internal working model is activated (diathesis). (2) The nature of the attachment style (secure, insecure-avoidant or insecure ambivalent) will then lead the appraisals made about the pain, one’s ability to manage it and other´s ability to provide support. (3) These appraisals will, in turn, determine the coping strategies, support-seeking behaviours and emotional states experienced. (4) Finally, these responses to the appraisals made will have an effect on each one’s experience and adjustment to pain.^16^

Meredith, Ownsworth and Strong^16^ specifically propose that those individuals with an insecure attachment will be more likely to develop chronic pain and less likely to manage it efficiently. This is due to the tendency individuals with insecure attachment have to present less adaptive appraisals (eg, higher catastrophizing, lower self-efficacy, etc.) and coping strategies (eg, emotion focused coping, denial coping, etc.).^16^

Different types of evidence have corroborated the proposals of this model.^75–80^ Overall, the theory is one of the best constructed ones, with explanations of aetiological factors and the different variables associated with them. However, although the theory is solid and supported by the evidence, the effect sizes are sometimes small^81^ and some contradictory findings have been encountered.^82^ Many potential explanations could be given for this. To begin with, we must consider that attachment is not an easy construct to measure and different methods may lead to different estimations.^83^ Furthermore, changes of attachment style could occur through the lifetime^84^ which presents numerous challenges for testing the model. The effects of attachment could be a consequence of the current attachment style or the attachment style on a critical developmental period, and if both do not match, identifying effects on chronic pain development could be challenging. To improve the quality of the model, future research could explore the temporal dynamics of the relationship between attachment and pain, perhaps through longitudinal studies. Another element that would increase the quality of the model would be the exploration of biological correlates of the proposal. Evidence already exists linking attachment with different neuroendocrine alterations.^85^,^86^ The inclusion of these elements in the model could be a way to complement the proposal and perhaps give way to the study of new methods of measurement and treatment.

Finally, we must consider that factors other than attachment will have an effect on chronic pain vulnerability. Consequently, to identify the relative importance of the proposals of the model, it would be interesting to compare them alongside other vulnerability factors. This would allow the identification of the relative weight attachment presents in comparison with other variables, and therefore, the value of their inclusion in clinical settings.

A summary of the evaluation criteria results can be observed in Table 2, and the full application of the evaluation criteria on the model can be seen in Table 7.

**Table 7:** Evaluation of the Attachment-Diathesis Model of Chronic Pain^19^

Model evaluation: The Attachment-Diathesis Model of Chronic Pain^16^	
**Clarity**	
*Does the model propose a specific set of variables that can be measured?*	
Yes	Attachment is a highly studied variable with multiple tools that allow its measurement
**Depth**	
*Does the model propose the aetiological factors that lead to the development of the proposed set of variables?*	
Yes	The factors that result in the development of a specific attachment style are well known and defined.
*Does the model provide a mechanistic explanation of chronic pain vulnerability?*	
Yes	The model provides the mechanisms responsible for the development of chronic pain vulnerability with a psychological and social angle.
**Whole system approach**	
*Does the model take a whole system approach?*	
No	The model presents mostly a psychological focus.
**Evidence**	
*What is the level of evidence provided to support this model?*	
Grade C	Cross-sectional research has encountered that fibromyalgia patients,^79^ somatoform pain disorder patients,^78^ chronic headache patients^80^ and children affected by migraines^75^ present higher rates of insecure attachment than healthy controls. Cohort studies show with small effect sizes that attachment insecurity is a significant predictor for the development of disability after whiplash injury.^81^ Nevertheless, other cross-sectional studies in whiplash associated disorder have found that attachment style is a vulnerability to develop PTSD and somatisation after an accident; however, they did not find a significant effect of attachment on pain and disability.^82^ Attachment insecurity has also shown to be a predictor of disability levels in migraine patients participating in a cross-sectional study.^87^ In a cross-sectional study, correlations between attachment style and different pain measures have also been encountered in children.^88^ Furthermore, as proposed by the model, an association between insecure attachment and appraisals has been encountered by a cross-sectional study, as well as a correlation between these variables and pain catastrophizing and distress.^77^ Moreover, in other cross-sectional studies, an association between attachment and coping strategies has been observed,^89^ as well as an association between anxious attachment and pain catastrophizing, fear of pain and hypervigilance.^90^ The model has been corroborated through a Structural Equation Modelling approach in a cross-sectional sample of adolescents and young adults with a history of childhood functional abdominal pain.^76^ In summary, research seems to support the proposals made in this model. The evidence associated is mostly of cross-sectional nature without control groups (level 4). A couple of cohort studies have also been performed although the results of these have been conflicting.

#### The Personality Hypothesis of Chronic Pain

The idea that a certain type of personality is more likely to experience chronic pain has been discussed in the pain literature for more than a century. Possibly, one of the first formal proposals of this link was made by psychoanalytic researchers and their theories surrounding the “hysteric” personality.^17^ Since then, research on different personality traits and chronic pain vulnerability has bloomed with variable results. The most common link is perhaps the association between neuroticism (a personality trait characterized by high emotional negativity) and the presence of clinical pain^18^,^19^ or neuroticism and psychological factors associated with pain, such as fear avoidance.^91^ Others have proposed that chronic pain vulnerability is driven by the presence of high harm avoidance (a temperament trait that is hypothesized to be dependent on biological traits) and low self-directedness (a character trait defined as the ability to self-adjust to achieve goals, that is hypothesized to be dependent on learning).^20^ In summary, certain personality traits that favour avoidance and/or catastrophizing could be a predisposing factor for chronic pain, although the effect sizes are small.^18^

In summary, evidence regarding the link between some personality factors^18^,^19^ and chronic pain vulnerability is present in the literature. However, some doubt has been cast into its validity with authors proposing that chronic pain might be what causes changes in personality^92^ and not the other way around. Evidence in this area is conflicted and characterized by small effect sizes.

Finally, social implications associated with the model call for a cautious analysis and application of this model in clinical practice. To begin with, we must consider that the ideas of the model have been around since the 19th century, which could increase the risk of dogmatic acceptance of the model. Furthermore, it is important to note that the initial ideas that inspired this model (hysteric personality) have been associated with the mistreatment of women in healthcare through history^93^ and seem to still have a discriminatory impact even in today’s medical settings.^94^

Consequently, although we cannot disregard the research that finds a link between some personality factors and pain, and it is important to point out that most research in the area has advanced from the Freudian view of chronic pain; social responsibility calls for a critical and cautious appraisal of the model, especially if we ever intend to bring it back to clinical practice. A summary of the evaluation criteria results can be observed in Table 2, and the full application of the evaluation criteria on the model can be seen in Table 8.

**Table 8:** Evaluation of the Personality Hypothesis of Chronic Pain

Model Evaluation: The Personality Hypothesis of Chronic Pain		
**Clarity**		
*Does the model propose a specific set of variables that can be measured?*		
No		Different authors propose different variables, there is no consensus.
**Depth**		
*Does the model propose the aetiological factors that lead to the development of the proposed set of variables?*		
Yes		Some proposals provide aetiological factors to the origins of the central variables (such as temperament or learning), others do not.
*Does the model provide a mechanistic explanation of chronic pain vulnerability?*		
Yes		Some proposals present descriptions of the proposed mechanisms, other proposals fail to do so.
**Whole system approach**		
*Does the model take a whole system approach?*		
No		Although in some cases mention of possible biological aetiological factors are made, this proposal takes mostly a psychological approach.
**Evidence**		
*What is the level of evidence provided to support this model?*		
Grade D	Some cross-sectional studies have found an association between neuroticism and chronic pain^18^,^19^ and pain intensity^95^ There is evidence supporting the relationship between higher rates of certain personality traits and other variables that have been associated to chronic pain vulnerability such as attentional bias in chronic pain patients or fear avoidance.^91^,^96^ In another cross-sectional study, neuroticism has been shown to act as a mediating variable between pain catastrophizing and pain vigilance and severity. This indicates that personality may affect pain by altering other intermediate variables.^97^ There seems to be a higher prevalence of personality disorders in chronic pain populations^98^ and cross-sectional research shows deviations from normal personality scores without reaching the levels of personality disorders.^91^ In summary, although some evidence exists indicating a possible link between personality and chronic pain vulnerability; results are still inconclusive, with small effect sizes and mostly based on cross-sectional research. Furthermore, it has been proposed through neuroimaging research that changes in personality could be a consequence of chronic pain^92^ and not only a vulnerability factor. Consequently, the evidence associated with the model is mostly of level 4 but with some contradictory or weak results.	

#### Psychological Variables and Comorbidities

Finally, although they are not part of a model, plenty of research on chronic pain vulnerability is centred on the different psychological variables and comorbid disorders that may prompt, accelerate, exacerbate or accompany the development of a pain condition.^99^ Sometimes, these studies will be framed in the analysis of one of the previously presented models;^33^ other times, they will be part of an independent correlation study.^100^ On the other hand, when it comes to comorbid disorders, chronic pain has been associated with several mental health and physical health issues. Associations with depressive and anxiety disorders,^101^ sleep disorders,^102^ obesity^103^ and coronary disease^104^,^105^ have been encountered by many researchers. The associations proposed are mostly bidirectional and therefore, lead to the suspicion of common vulnerabilities.

It is difficult to assess the quality of these proposals since they are all framed independently and not under the same unifying theory, making it a challenge to guide investigations in the area. Consequently, the development of a synthesizing model in which the common vulnerabilities and aetiological factors for the different disorders are presented alongside the mechanisms that lead comorbidity would be a beneficial addition to the research field.

A summary of the evaluation criteria results can be observed in Table 2, and the full application of the evaluation criteria on the model can be seen in Table 9.

**Table 9:** Evaluation of Psychological Variables and Comorbidities Evidence

Model Evaluation: Psychological Variables and Comorbidities	
**Clarity**	
*Does the model propose a specific set of variables that can be measured?*	
No	The variables studied vary between studies
**Depth**	
*Does the model propose the aetiological factors that lead to the development of the proposed set of variables?*	
No	● Studies are normally centred in uncovering the existence or lack of existence of a correlation between the psychosocial variable or disorder of interest and chronic pain.
*Does the model provide a mechanistic explanation of chronic pain vulnerability?*	
No	Studies are normally centred in uncovering the existence or lack of existence of a correlation between the psychosocial variable/ disorder of interest and chronic pain.
**Whole system approach**	
*Does the model take a whole system approach?*	
No	Although in some cases there is mention of possible biological factors, these proposals take mostly a psychological approach.
**Evidence**	
*What is the level of evidence provided to support this model?*	
Grade B	The support collected varies between proposals. Since multiple variables and conditions are studied it is difficult to address the support of all of them in general. Some variables show a greater influence on chronic pain than others. Several cohort studies^33^,^105^ and meta analyses^99^,^104^ can be found supporting the correlation, setting the overall level of evidence to level 2.

#### The Developmental Approach to Chronic Pain Vulnerability^24^

This model proposes chronic pain as a consequence of alterations in healthy developmental processes, understanding vulnerability as a phenomenon that can be influenced by the different changes (biological, psychological and social) that occur through the lifespan. Particularly, the authors put the focus on the paediatric development of nociception and pain, long-term effects of paediatric pain and biological, psychological and socioenvironmental aging changes. It is proposed that if alterations occur in these processes, they could lead to chronic pain vulnerability.^21^

This idea shows good promise as a concept that could help understand the aetiology and mechanisms of chronic pain, and in fact, supporting evidence for these principles can be found in the literature. For instance, research has found that childhood pain is a predictor of adulthood pain^106^ and that neonatal care experiences alter the nociceptive system of children.^107^,^108^ Moreover, research has also pointed out at a potential developmental effect of parental chronic pain that influences the development of chronic pain through childhood.^109^ On the same line, the previously pointed out research regarding the attachment model^75–80^ and childhood trauma^54^,^110^,^111^ can also be interpreted under this model as a confirmation of the effects that developmental processes or disruptions to them can have on long-term chronic pain vulnerability. Finally, literature studying the changes in aging associated with chronic pain has also encountered supporting outcomes, with results indicating that aging is associated with changes in endogenous pain modulation.^112^

Although the evidence associated with the model is positive and the prospects of finding aetiological and mechanistic explanations through this approach are promising, the model still presents some limitations. These limitations are similar to the ones encountered by the bio-psycho-social model of chronic pain, namely the breath of the model impedes the identification of specific variables that could be measured in clinical settings. Consequently, future research could focus on identifying the key processes that lead vulnerability, in order to provide a more specific set of characteristics that could be used in practice to identify vulnerability.

A summary of the evaluation criteria results can be observed in Table 2, and the full application of the evaluation criteria on the model can be seen in Table 10.

**Table 10:** **Evaluation of the Developmental Approach to Chronic Pain Vulnerability Evidence**^24^

Model Evaluation: The Developmental Approach to Chronic Pain Vulnerability	
**Clarity**	
*Does the model propose a specific set of variables that can be measured?*	
No	The variables studied vary between studies. The model is not characterized by a number of set variables, alternatively provides a compendium of different hypotheses.
**Depth**	
*Does the model propose the aetiological factors that lead to the development of the proposed set of variables?*	
Yes	A general aetiology is provided: Disruption of the healthy developmental processes. However, under the umbrella of this aetiological explanation numerous factors could be included. Consequently, a general principle is provided but this lacks specificity.
*Does the model provide a mechanistic explanation of chronic pain vulnerability?*	
Yes	Several hypotheses are provided, some of which present mechanistic explanations, however, no specific mechanism is proposed as the main contributor to vulnerability.
**Whole system approach**	
*Does the model take a whole system approach?*	
Yes	The model includes mentions to psychological, biological and social aspects.
**Evidence**	
*What is the level of evidence provided to support this model?*	
Grade B	The different hypotheses of the model are supported by the evidence. A cohort study observed childhood pain as a predictor of chronic pain in adulthood.^106^ Parent behaviour pointed out as a factor by the model has also shown to influence pain development. For instance, in a cross-sectional study, it was observed that children of parents with chronic pain have a higher chance of developing chronic pain themselves.^113^ Changes in pain modulation associated with aging have also been found in a cross-sectional study^112^ Since the model serves more as an approach to understand vulnerability more so than providing specific variables, it is difficult to evaluate the specific level of evidence. Different proposals included in the model will present different levels of evidence (for instance the evidence associated with childhood pain is stronger than the evidence regarding aging and pain modulation). All in all, a lot of the evidence associated with the model is found in cross-sectional and occasionally in cohort designs. Grade B has been granted due to some proposals of the model such as the effect of childhood pain reaching the required criteria. Nevertheless, it must be considered that not all proposals present this level of evidence.

## Discussion

The models presented above are grounded in a large amount of research that identified variables that interact in the onset and maintenance of chronic pain. Table 2 provides a summary of the model evaluations. We found that most of the current models provided excellent specific measures and that most were also supported well by empirical evidence, indicating their validity to be used in practice. Particularly, the fear avoidance model,^9^,^10^ the diathesis-stress model,^8^ the shared vulnerability^11^ and mutual maintenance models,^12^ and the attachment diathesis model^77^ seem to be strongest on these points, whereas the personality hypothesis was the weakest. Nevertheless, all models present some limitations. Particularly, the focus of most of the models is not directed to study in-depth the aetiology of the vulnerability and the mechanisms that lead to it. Furthermore, most models take a psychological approach with little focus on biological and social factors and on the interactions between bio-psycho-social variables. In summary, the presented models do an excellent job of identifying the psychological profile for chronic pain vulnerability but do not delve into the causes of this profile or its biological correlates.

This is an understandable approach when considering the multifactorial nature of chronic pain and how difficult it can be to create an all-encompassing theory. In fact, the limitations of including multiple variables can be observed when evaluating the bio-psycho-social model of chronic pain. In this model, in contrast to the other models, a great focus is given to presenting a whole system approach and identifying aetiological and mechanistic factors. However, due to the breath in scope, the model falls short in giving specific variables that can be measured in a clinical setting to estimate individual risk.

Based on the results of this evaluation, it is clear that a middle ground between both approaches (whole-system vs specific factors) is hard to obtain. Nevertheless, certain steps can be taken based on our observations to increase the quality of the models and aid clinicians and researchers in the application and improvement of the current knowledge. To begin with, as we have mentioned throughout the text, some key variables in the more specific models (eg, anxiety sensitivity) have been questioned as to their real influence on chronic pain development. Therefore, a re-analysis of the relative importance of the different variables would be a beneficial step to take by researchers. Furthermore, another way to improve the quality of the more specific models might be to directly compare the models in terms of the predictive value of the variables that compose them. For instance, research could collect data on the main variables of the diathesis-stress model, fear avoidance model and the attachment-diathesis model, and then the predictive value of each one of the models could be compared to establish which would be the most useful for clinicians. Complementarily, a factor analysis could be performed with the variables from different models in order to identify overlapping or equivalent constructs between models, or even to establish which combination of variables offers the most information regarding vulnerability to chronic pain. Finally, another way to improve the quality of the models would be to tackle the lack of attention of each one of the models to aetiological and mechanistic factors. In this way, each one of the models could try to identify the most likely aetiological factors contributing to their key variables. For instance, the diathesis-stress model could explore the influence of traumatic life events or certain genetic predispositions on the variables of anxiety sensitivity and pain catastrophizing. It would be particularly interesting to study the mechanistic relationship between psychological and biological findings as this has been suggested to be a way to move forward in identifying the causal narrative of a disease.^114^

Furthermore, a mechanistic focus would encourage the use of experimental designs that go further than the observational level, to build evidence at a higher level than those currently related to the models. A way to explore the mechanistic and causal relationships may be through Randomized Controlled Trials (RCTs) in which the intervention modifies suspected causes and mechanisms in order to test whether chronic pain is prevented by specifically targeting those causes/mechanisms. For instance, an RCT in which attachment-based therapy vs other psychological treatment was delivered to a chronic pain sample would serve to whether insecure attachment is influencing pain. Nevertheless, since these may be costly and difficult to carry out, alternatives to this option exist. To begin with, instead of targeting the mechanisms of a disease through RCT, measures of a suspected mechanism could be included in RCTs exploring the effectivity of different treatments and their influence on treatment success could be explored this way. Furthermore, an alternative to RCTs has been proposed in the use of computational designs such as Bayesian predictive models.^115^ Particularly, in this type of design probabilistic models can be created that try to replicate the process of pain perception that the brain performs. By evaluating the ability of the models to predict real outcomes, some light can be shed into the processes that the organism is undergoing. For instance, to study the hypothesis that attachment could be influencing chronic pain vulnerability, the attachment score could be included in a Bayesian model as a prior of the weight placed on the influence of psychological states on pain perception within an experimental task or by using general pain rating scores of chronic pain. Then the predictive ability of this model could be compared to another without the inclusion of this informative prior.

Furthermore, it would also be valuable to perform prospective cohort studies to examine the prognostic factors associated with the development and maintenance of pain.

This is the first work that evaluates the quality of the different conceptual models proposed for chronic pain vulnerability. We consider this an important step to ensure both the avoidance of dogmatic acceptance of old theories and to identify the points that need further attention in future research. Nevertheless, it is also important to note that, although necessary, this work presents certain limitations. Particularly, it must be considered that the evaluation criteria have been developed based on what the literature has identified as the goals of conceptual models, but other important quality criteria could exist that have not been considered here.

Finally, it is important to mention the practical applications of the current models. As it can be seen through the evaluation, although the models show room for improvement, most of them already excel in their clarity, meaning that they provide specific factors that would be easy to measure in clinical settings. Considering this practicality and that the evidence gathered mostly supports the proposals of the models, it would already be feasible for the model variables to be used to identify those at a higher risk of developing chronic pain and therefore providing them with appropriate preventative help. These types of preventative efforts would be most suitable for patients that suffer a traumatic injury since a lot of the current evidence is based on pain chronification in this population. This way, clinical services could measure the vulnerability variables identified by the models and provide psychological help or advice to those who show high vulnerability. Regarding the specific model variables that should be used, perhaps the first option would be the ones presented by the fear avoidance model, since, of those models with good clarity, this one presents the highest quality and quantity of evidence.

In conclusion, current conceptual models for chronic pain vulnerability provide good descriptive evidence of the relationship between different psychological variables associated with chronic pain vulnerability. Nevertheless, in order to improve these models and gain more insight into what is leading the vulnerability, it would be important to explore in further detail the potential causal factors and mechanisms maintaining the condition and doing so from a bio-psycho-social approach.
